# Peruvian Bark

**Published:** 1874-12

**Authors:** 


Peruvian Bark.—It is well known that from cinchona or Peruvian bark is extracted the active salt called quinine. Medical men have looked upon quinine as the only valuable ingredient in the bark, and have treated the spent bark as so much inert woody substance. It now appears, from a statement made by Dr. Hamilton, in the *India Medical Gazette*, that the bark has a power which quinine has not. Careful experiments show that cinchona is competent to ward off malarial fevers, against which the quinine proves of little or no avail. This theory of the superiority of the bark is also sustained by the *London Lancet*, which warmly urges its use as an anti-periodic. It is therefore probable that the bark contains some valuable principle which is not obtained by the acid employed in extracting the quinine.

